# Supplementary material for: Dileucine ingestion, but not leucine, increases lower body strength and performance following resistance training: A double-blind, randomized, placebo-controlled trial
Source: PLoS One. 2024 Dec 31;19(12):e0312997. doi: 10.1371/journal.pone.0312997 (PMC11687731; doi:10.1371/journal.pone.0312997)
Supplement: S1 File — (PDF) [file pone.0312997.s001.pdf]

Submission Type: Initial Date: 3-4-2024

IRB #: IRB-21-64
Title: A Pilot Study to Examine Changes in Resistance Training Adaptations After Leucine and Dileucine Ingestion
Creation Date: 11-30-2020
Status: Review Complete
Principal Investigator: Chad Kerksick

Welcome

Welcome to the Lindenwood University IRB Application. This application will guide you through the process of submitting your proposed research involving human subjects to the IRB (Institutional Review Board) for review.

This application is an opportunity to consider the ethical dimensions of your research, while also allowing you to consider a few of practical aspects of your research protocol in more detail. We looking forward to partnering with you in this process of considering the rights, welfare, and dignity of all participants in the research process.

--

As you can see from the tabs along the left hand side, this application is set up to track with you through the life-cycle of a research protocol. Each tab addresses a new component or stage of the research process. Each tab also will connect you with a different ethical criterion used by the IRB to review your proposal.

Each tab represents a different ethical step in considering the implications of your research for participants and others. Please keep the following general concepts in mind as you complete the application:

- Are all risks to subjects minimized?
- Are all risks reasonable in relation to the potential benefit?
- Are all subjects selected with equity and respect?
- Will you talk to subjects and obtain their consent?
- Will you document the consent process?
- Will you monitor the safety of subjects?
- Will you protect the privacy of all subjects?
- Are there any special ethical considerations for vulnerable populations?

Basic Information

*required

1. **Who is the Principal Investigator?**

The Principal Investigator is the individual primarily responsible for ensuring all aspects of the research are conducted in compliance with all applicable policies and regulations. The Principal Investigator ensures the rights, welfare, and dignity of participants are protected. This person may be Faculty, Adjunct Faculty, Staff, Graduate Student, or Undergraduate Student.

Name: Chad Kerksick
Organization: Exercise Science
Address: 209 South Kingshighway , St. Charles, MO 63301
Phone:
Email: [redacted]

*required

2. **Who is the Primary Contact?**

The Primary Contact is the main administrative contact for the study. In many cases, the PI will also serve as the Primary Contact.

Name: Richard Stecker
Organization: Exercise Science
Address: 209 South Kingshighway , St. Charles, MO 63301
Phone:
Email: [redacted]

*required

3. **In what role is the PI conducting this research?**

✓ Faculty Researcher

Adjunct Faculty Researcher

Staff Researcher

Graduate Student Researcher

Undergraduate Student Researcher

Who are your Research Team Members?

List all individuals affiliated with Lindenwood University who will be engaged in human subjects research for the purposes of this project. ***Please do not list dissertation or thesis committee members unless they are directly engaged in human subject research activities.***

Name: Jessica Moon
Organization: Exercise Science
Address: 209 South Kingshighway , St. Charles, MO 63301
Phone:
Email: [redacted]

Name: Kayla Ratliff
Organization: Exercise Science
Address: 209 S. Kingshighway , St. Charles, MO 63301
Phone:
Email: [redacted]

Name: Kylie Walden
Organization: School of Health Sciences
Address: 209 S. Kingshighway , St. Charles, MO 63301
Phone:
Email: [redacted]

Name: Connor Gaige
Organization: Exercise Science
Address: 209 S. Kingshighway , St. Charles, MO 63301
Phone:
Email: [redacted]

Name: Anthony Hagele
Organization: School of Health Sciences
Address: 209 S. Kingshighway , St. Charles, MO 63301
4. Phone:
Email: [redacted]

Name: Johnathan Boring
Organization: Exercise Science BV
Address: 2600 W Main St. , Belleville, IL

Phone:
Email: [redacted]

Name: Petey Mumford
Organization: Exercise Science
Address: 209 S. Kingshighway , St. Charles, MO 63301
Phone:
Email: [redacted]

Name: Kyle Sunderland
Organization: Exercise Science
Address: 209 South Kingshighway , St. Charles, MO 63301
Phone:
Email: [redacted]

Name: Logan Orr
Organization: Exercise Science
Address: 209 S. Kingshighway , St. Charles, MO 63301
Phone:
Email: [redacted]

Name: Athena Viers
Organization: Exercise Science
Address: 209 S. Kingshighway , St. Charles, MO 63301
Phone:
Email: [redacted]

Name: Maycee White
Organization: Exercise Science
Address: 209 S. Kingshighway , St. Charles, MO 63301
Phone:
Email: [redacted]

*required

5. **Will an individual not affiliated with Lindenwood University be part of the Research Team?**

✓ No

Yes

*required

Do you or any of your Research Team Members have a Conflict of Interest related to this research?

6.

Please refer to the additional information in the "?" button for information about what constitutes a Conflict of Interest. The review of Conflicts of Interest by the Investigator Conflict of Interest Committee will occur in a process ancillary to the IRB application, to ensure the confidentiality of PI interests.

✓ No

Yes

*required

Where will your research take place?

7.

List all research sites and locations. You should list any specific location, such as a school, institution, or a specific classroom. An online, gaming, or virtual "space" counts as a research location and should be listed. If you are collecting secondary data from a database, please list the database or source.

Within the Exercise and Performance Nutrition Laboratory (EPNL) at Lindenwood University and Field House 110. Both of these sites are located within the same building (The Field House) and are approximately 15 seconds apart from one-another.

*required

Are any of these sites outside the United States?

8.

Please only answer "Yes" if your research will physically be conducted outside the United States, or data will be collected from sources outside the United States. Online surveying, social media, or similar virtual research spaces are not applicable to this question.

✓ No

Yes

*required

9. **Do any of these sites require permission or advanced notice?**

Schools, organizations, business, and similar environments often require permission prior to conducting research in their facilities. Researchers at Lindenwood University are required to ensure compliance with these local policies prior to conducting research. If a research site is subject to HIPAA, FERPA, or other applicable regulations for the collection of research data, the site authorization must provide affirmation of adherence to these regulations at each site.

✓ No

Yes

*required

10. **Will any research be conducted at sites external to Lindenwood University that have their own IRB?**

✓ No

Yes

*required

11. **Are you requesting the Lindenwood University IRB to rely on another IRB for Single IRB (SIRB) Review?**

✓ No

Yes

Your Funding Information

*required

12. **Will this research be supported by any funding?**

Please visit the LU Grants and Sponsored Projects *website for more information.*

No

✓ Yes

*required

12a. **Did you complete the Pre-Award Proposal Review Process?**

✓ Yes

No

*required

12b. **Please select your Sponsor.**

Please select "Unknown" if you do not see your Sponsor Code. The IRB will assist in creating a new Sponsor Code for your funding agency.

Name

-

- Name - A to Z
- Name - Z to A

Ingenious Ingredients, L.P.

*required

12c. **What is the name of the PI who is the prime awardee of the funding?**

Chad Kerksick, PhD

*required

12d. **What is the nature of the funding?**

✓ Direct

Indirect/Pass-Through

Subaward

Continuation/Renewal

*required

12e. **What is the Grant Title?**

A Pilot Study To Examine Changes in Resistance Training Adaptations After Leucine and Dileucine Ingestion

*required

12f. **What is the Project Period for the Funding?**

Approximately December 2020 - August 2020

Your Research Question

*required

What is your research question?

13. *Think of this section as your research "elevator pitch." Please briefly describe the question(s) or issues you are addressing with your research (limited to 100 words). You will be able to provide information on specific outcomes, hypothesis, or related analysis in a following question.*

This study seeks to compare the observed changes in resistance training adaptations after supplementation of isomolar (same volume of solution) amounts of leucine or dileucine in healthy resistance-trained men. This will provide a better understanding of supplementation with leucine versus dileucine over a 10-week period in regards to increasing muscular performance.

*required

How will you answer your research question?

14. *Please provide a brief summary of procedures you will use to collect or produce data (limited to 100 words). You will be able to provide more detailed protocol information in a following question.*

The study will be conducted using a randomized, double-blind approach with individuals being provided daily 2-gram doses of leucine, 2-gram doses of dileucine, or a placebo. Daily blinded supplementation will occur each day for a 10-week period of time while completing a heavy resistance training program. Participants follow the resistance training program for a total of 10 weeks. To assess the efficacy of the two different forms of amino acid supplementation on resistance training adaptations, changes in fat-free, lean, and fat mass will be determined using a 4-compartment body composition model. Skeletal muscle cross-sectional area will be assessed using ultrasound. Maximal strength, muscular endurance, and power will also be assessed. Participants will be required to provide weekly compliance and complete one supervised workout per week. After 2, 6, and 10 weeks of following the supplementation and resistance training regimens, participants will return to the laboratory to complete testing bouts consisting of identical assessments of body composition, muscular strength, muscular endurance, lower-body power, and anaerobic capacity. Adverse events related to each supplementation group will be recorded and assessed.

*required

What do you think are the most critical ethical issues with your research?

---

15. *Please provide one or two sentences describing what you think are the key ethical issues or concerns associated with your research. For example, your research poses significant risks to subjects, it may be difficult to obtain voluntary consent from participants, or you would like to interact with subjects who are vulnerable or often subject to coercion.*

Risks to subjects are reasonable in relation to anticipated benefits, if any, to subjects, and the importance of the knowledge that may reasonably be expected to result.

*required

Why is your research important, and how might your research findings contribute to scholarship or current study in your field?

---

16. *Please limit your response to 250 words. This response can include a summary or history of research in your question, issue, or area. You may attach 1-2 pages of bibliography or additional information as applicable.*

For the past decade, exceptional interest has been generated regarding the impact of the essential amino acid, leucine. Currently, it is well accepted that leucine operates as a key stimulator of protein synthesis in both animal and human research models. Further research has gone on to indicate that a minimum threshold of leucine exists within skeletal muscle before widespread activation of translational machinery occurs. In this respect, leucine doses of 1.7 – 3.8 grams have been reported to be the optimal range of leucine to activate muscle protein synthesis (Tipton, Ferrando et al. 1999, Paddon-Jones, Sheffield-Moore et al. 2004, Norton 2009). Different protein sources are widely known to have varying amounts of leucine, the branched-chain amino acids and the other essential amino acids (Phillips, Tang et al. 2009, Joy, Lowery et al. 2013). Dileucine is a unique peptide that consists of two leucine molecules bonded together. Currently, no published literature is available that has reported on the resistance training adaptations after supplementing with 2 grams of leucine or dileucine for a period of 10 weeks. These data are important for multiple reasons. For starters, studies have linked changes in leucine concentration with the anabolic potential of a given source of amino acids (Norton 2009). Furthermore, the speed as well as the magnitude of changes in leucine concentration may operate as key variables that explain the anabolic potential of dileucine.

Attach additional information:

16a.

REFERENCES.docx

*required

17. **How will you measure your results or analyze your research data?**

Please include a list of your hypotheses, study aims, and/or intended study outcomes. Also include a brief description of any statistical analyses you may use to meet these outcomes. It is important to be clear about any intended outcomes, as this information is vital in assessing the prospective benefit of a research proposal.

Specific Aims:

1) In comparison to daily leucine supplementation, we aim to examine the impact of dileucine on the observed changes in strength, power, and body composition after eight weeks of supplementation and heavy resistance training.

2) In comparison to placebo supplementation, we aim to examine the impact of leucine and dileucine on the observed changes in strength, power, and body composition after eight weeks of supplementation and heavy resistance training.

Independent Variable

Supplementation Status:

- 2 grams Dileucine (n=12)
- 2 grams Leucine (n=12)
- Placebo (n=12)

Dependent Variables

Primary Endpoints:

- Fat-free mass
- Leg press 1RM
- Bench press 1RM
- Maximal isometric mid-thigh pull force production

Secondary Endpoints:

- Fat mass
- Lean mass (DEXA)
- % Body Fat (DEXA)
- Total, extracellular, and intracellular body water
- Skeletal muscle cross-sectional area via ultrasound
- Bench press repetitions to fatigue
- Leg press repetitions to fatigue
- Peak anaerobic power
- Mean anaerobic power
- Rate of fatigue
- Maximal concentric rate of force development

STATISTICAL ANALYSIS:

All analyses will be completed using Microsoft Excel and the Statistical Package for the Social Sciences (v23; SPSS Inc., Chicago IL). Before any statistical tests are performed, data will be analyzed for normality, skewness, and kurtosis. All non-normal data will be log-transformed prior to analysis. For all statistical tests, data will be considered statistically significant when the probability of a type I error is 0.05 or less. Primary endpoints for this investigation will considered to be the delta (Week 10 – Week 2) value for DEXA fat-free mass and leg press 1RM. Secondary endpoints will be delta (Week 10 – Week 2) values for DEXA fat, DEXA lean and DEXA % fat along with bench press 1RM, bench press repetitions to fatigue, leg press repetitions to fatigue, leg press volume, peak anaerobic power, mean anaerobic power, and rate of fatigue. 2 x 2 mixed factorial (group x time) ANOVA with repeated measures on time will be used to determine any statistically significant differences for time and group main effects and group x time interaction effects. Additionally, 95% confidence intervals will be constructed of the observed changes for each group to assess within and between-group changes. All data will be presented as means ± standard deviations.

*required

What are your study procedures?

Describe all study procedures and protocol elements in chronological order. Be sure to include (no word limit):

- *Everything you will ask participants to do, and the duration and frequency of these protocol elements. (Lists, tables, or charts may be helpful for complicated studies.)*
- *Details regarding all procedures, interventions, and methods of data collection.*
- *Any study design elements such as randomizing, blinding, cross-over, etc...*
- *Description of any substances, devices, or equipment used during the study.*
- *If applicable, specify whether each procedure will occur for research purposes or as part of standard of care.*

Experimental Design

The study will be conducted using a randomized, double-blind approach with individuals being provided daily 2-gram doses of leucine, 2-gram doses of dileucine, or a placebo. Daily blinded supplementation will occur each day for a 10-week period of time while completing a heavy resistance training program. Dosing on workout days will occur within 60 minutes of completing each workout while doses on non-workout days will be ingested with their morning meal. On workout days, assigned dosing will be ingested within 60 minutes of completing their workout and on-days when study participants complete a workout at university facilities, the assigned supplement will be consumed in front of a research team member. The resistance training protocol will follow a linear periodization, split-body resistance training design consisting of two upper body and two lower body workouts each week resulting in each muscle group being trained twice each week. To minimize the influence of training experience and any learning effect, individuals with at least 12 months of resistance training history will be recruited. Participants follow the resistance training program for a total of 10 weeks. To assess the efficacy of the two different forms of amino acid supplementation on resistance training adaptations, changes in fat-free, lean, and fat mass will be determined using a 4-compartment body composition model. Skeletal muscle cross-sectional area will be assessed using ultrasound. Maximal strength will be assessed by determining one-repetition maximums (1RM) for the bench press and leg press exercises in addition to completion of an isometric mid-thigh pull. Muscular endurance will be assessed by determining the maximal number of repetitions completed using 80% of the 1RM (i.e. multiple-repetition maximum [RTF]) determined at baseline. Lower-body muscular power will be assessed using countermovement jumps and anaerobic capacity using a Wingate anaerobic capacity test. Participants will also be provided nutritional recommendations to ensure adequate energy and macronutrient consumption to facilitate positive training adaptations and eliminate any potential influence of differing dietary intakes. Participants will be required to provide weekly compliance and complete one supervised workout per week. After 2, 6, and 10 weeks of following the supplementation and resistance training regimens, participants will return to

the laboratory to complete testing bouts consisting of identical assessments of body composition, muscular strength, muscular endurance, lower-body power, and anaerobic capacity. Adverse events related to each supplementation group will be recorded and assessed.

Familiarization and Entry (Visit 1)

At the first lab visit, participants will receive an informed consent document and will be required to give full consent before proceeding. Upon providing consent, participants will provide personal and emergency contact information before completing a healthy history and exercise history form. Study participants will receive a dietary log and will be asked to record their nutrient intake over a 3-day period (2 weekdays and 1 weekend day) for assessment of daily energy and macronutrient intake. Participants will then have initial height and body mass measurements completed. The body mass assessments taken at this time will be used as a comparison to their Visit 2 body mass to ensure participants are weight stable. To conclude this visit, participants will complete the 1RM protocol on both the bench press and leg press exercises and will complete the mid-thigh pull test, countermovement jump (CMJ) protocol and Wingate anaerobic capacity (WanT) protocol. If screened eligible due to adequate baseline strength, study participants will then be oriented to the resistance training program they will be required to follow throughout the study.

This orientation will consist of a discussion with a research team member on how to properly progress the loads they will use during each workout in addition to completing their training log in addition to discussion what dietary requirements are expected throughout the protocol. Participants will then complete a strength orientation session where an 8 to 10-repetition maximum load will be determined for all exercises they plan to utilize to begin the workout program. In addition, participants will be instructed on how to use their workout card to properly progress loads used during each workout. All participants will be required to log completion of each workout. This study visit is estimated to take 90 minutes to complete.

To document and facilitate compliance, participants will be required to log completion of each workout, send a picture of them in the gym next to exercise equipment, and schedule at least one workout per week to be completed on campus in our laboratory facilities. If individuals want to complete more workouts each week at the facilities housed at our university, they will be accommodated.

Baseline Testing (Visit 2)

At least three days after Visit 1 to allow for completion of their food record, participants will return to the lab to complete their first testing session and begin following the resistance training and supplementation regimens. Participants will first have their body mass assessed before providing a urine sample and completing their body water, DEXA assessments (i.e. 4-compartment assessment), and muscle thickness via ultrasound. This scan will be done between the hours of 0600-1000 following an overnight fast and hydration levels will be standardized through the use of urine specific gravity measurements using a hand-held refractometer. Participants will be sent a hydration protocol to follow 24 hours prior to their visit to help ensure adequate hydration levels. This protocol will instruct the participants how much water they should be consuming throughout the day. Prior to testing, participants will complete the same standardized warm-up consisting of 5 minutes of light cycling, 10 body weight squats, 10 body weight walking lunges, 10 dynamic walking hamstring stretches (straight-leg march), and 10 dynamic walking quadriceps stretches. After the warm-up,

participants will complete five CMJ with their hands on their hips. One minute of rest will be observed between each CMJ. Participants will have their maximal voluntary isometric contraction (MVC) completed using an isometric mid-thigh pull. Three attempts will be made and one minute of rest will be observed between each test. Participants will then have their 1RM determined using the leg press exercise. Three minutes of rest will be provided after 1RM is determined and each participant will perform repetitions to failure using 80% of their previously determined 1RM to determine their repetitions to fatigue (RTF). An additional 3-minute break will be observed and then bench press 1RM and RTF will be completed using identical procedures as the leg press exercise. After observing a three-minute rest period after completion of the bench press RTF participants will complete a Wingate using a load that is equivalent to 7.5% of their body mass to measure anaerobic capacity (Lode Excalibur Sport, Lode, Netherlands). Participants will then complete an education session with a research team member to outline the dietary goals they are required to meet throughout this study. This study visit will take approximately 90 minutes to complete.

In a randomized, double-blind, placebo-controlled fashion, participants will be matched according to their Visit 2 fat-free mass to ingest either two grams of dileucine, two grams of leucine, or a placebo (rice flour). All supplements will be provided by Ingenious Ingredients, L.P. (Lewisville, TX). On days where research participants will complete the exercise program, they will be required to consume their daily dose within 60 minutes of completing their workout. When the workout is completed at our facility, supplement ingestion will be monitored by a research team member. On non-training days, participants will consume their assigned dose with their morning meal. Participants will be given enough supplement for one week at a time as participants will be required to schedule and complete one workout per week under the direct supervision of a research team member. During these weekly workouts and check-in visits, participants will get more supplement, discuss their dietary compliance and ensure completion of their workout cards. Participants will continue to follow the prescribed resistance training program for the entire 10 weeks of the study protocol.

Resistance Training & Supplementation Phase (Visits 3 and 4)

After completing two weeks of resistance training (8 total workouts), participants will return to the lab at a similar time (0600 – 1000 hours) for completion of an identical bout of testing. Prior to this visit, participants will complete another three-day food record and discuss with research team members about their compliance to the diet program. Participants will also have their body mass recorded. Participants will then donate a urine sample to assess hydration before completing a body water, DEXA , and ultrasound assessment. Using identical procedures as before, participants will have their CMJ performance, maximal strength (1RM and isometric mid-thigh pull) and endurance (maximal repetitions at 80% 1RM [RTF]) assessed before completing a Wingate anaerobic capacity test (WanT). New 1RM values will be used to re-assign loads during the following resistance training period. This visit will take approximately 90 minutes to complete.

Final Study Visit (Visit 5)

Approximately ten weeks after beginning the resistance training (40 workouts; a minimum of 36 required or 90% compliance) and supplementation protocol, study participants will report back to the lab for their final study visit during a similar time of day (0600 – 1000 hours). In an identical fashion as previously completed, participants will observe an overnight fast, ensure completion of all food and training records, return any remaining supplements and have their body mass, hydration, body water, body composition (DEXA), ultrasound, countermovement jump, maximal strength (bench

press 1RM, leg press 1RM, and isometric mid-thigh pull), muscular endurance (repetitions to failure using 80% [RTF] of their Visit 2 1RM for both exercises) and anaerobic capacity (WanT) determined. This visit is estimated to take 90 minutes to complete.

Table 1: Outline of testing for the 10-week study.

Procedure	Pre-Study (Visit 1)	Week 0 (Visit 2)	Week 2 (Visit 3)	Week 6 (Visit 4)	Week 10 (Visit 5)
Resistance Training Protocol		⟷	⟷	⟷	⟷
Supplementation Protocol		⟷	⟷	⟷	⟷
Review & Sign Consent	X				
Screening (Health, Medical, Exercise History)	X				
Height and Weight	X	X	X	X	X
Heart Rate / Blood Pressure	X	X	X	X	X
Food Record*	X	X	X	X	X
Randomization into Group	X				
Body Composition		X	X	X	X
One-Repetition Maximum (1RM)	X	X	X	X	X
Isometric Mid-Thigh Pull	X	X	X	X	X
Countermovement Jump	X	X	X	X	X
Muscular Endurance		X	X	X	X
Anaerobic Capacity	X	X	X	X	X
Dietary Protocol*		X	X	X	X
Resistance Training Program*		X	X	X	X
Exercise Training Compliance*		X	X	X	X
Review Supplementation Protocol*	X	X	X	X	X
* = Will occur weekly online and during in-laboratory study visits					

Procedures

Body Mass

Body mass weight will be measured (Tanita BWB-627A, Tokyo, Japan) and recorded to the nearest ± 0.1 kg upon arrival at all study visits. The recorded body mass during visit 1 and visit 2 will be compared to ensure the participant is weight stable and any participant whose body mass changes by more than 3% during this time will be excluded from participation. In addition, body weight will be assessed to compare changes seen after completion of the supplementation and resistance training protocols.

Body Composition Assessment

To achieve the highest levels of sensitivity and accuracy, a four-compartment body composition model will be employed according to the methods of Wilson et al. 2013 (Wilson, Fan et al. 2013) and Smith-Ryan et al. 2017 (Smith-Ryan, Mock et al. 2017). Body volume and bone mass will be assessed using a dual-energy x-ray absorptiometry (DEXA) scan (Hologic QDR Discovery A, Waltham, MA) on four separate occasions (visits 2, 3, 4, and 5). In addition, the lean tissue component will be estimated to quantify lean and fat-free body masses for each participant. Participants will be required to observe an overnight fast in order to ensure an accurate determination of body composition and hydration will be standardized using urine specific gravity measurements by a urine refractometer. All DEXA scans will occur during the hours of 0600 and 1000 by graduate assistants or faculty trained in DEXA administration and analysis. Prior to each

day of testing, the device will be calibrated according to manufacturer guidelines. All scans will be completed according to device specifications.

Urine Specific Gravity

Using a handheld refractometer and prior to each DEXA, participants will donate a mid-stream urine sample for determination of their urine specific gravity and assessment of hydration status. Using a transfer pipet, approximately 500uL of urine will be transferred onto the refractometer and compared against a standardized evaluation scale. Urine specific gravity values above 1.020 will be deemed as a dehydrated sample. Participants will be required to remain in the lab consuming water until they produce a urine specific gravity sample that is below 1.020. To encourage optimal hydration upon arrival, participants will be given instructions to follow to ensure proper hydration levels are achieved.

Body Water Assessment

Total, intracellular, and extracellular body waters will be assessed using an InBody 570 bioelectrical impedance analyzer on four separate occasions (visits 2, 3, 4, and 5). Participants will be required to observe an overnight fast to ensure an accurate determination of body composition. All BIA assessments will occur between 0600 – 1000 hours by trained research personnel. Prior to each day of testing, the device will be calibrated and all assessments completed according to manufacturer guidelines.

Ultrasound Assessment

The Ultrasound (GE Doppler Ultrasound Scanner) will be used on four separate occasions (visits 2, 3, 4, and 5) to complete muscle thickness measures with a 3 to 12 MHz multi-frequency linear phase array transducer (Logiq S7 R2 Expert; General Electric). The ultrasound wand will be placed on the participant’s vastus lateralis (outer thigh muscle) midway between the iliac crest (hip crease) and patella (knee) of the dominant leg while the participant is lying in a supine (on their back) position on an exam table. During the assessment, the research team member will be able to determine the thickness of the participant's thigh.

Maximal Strength - One-Repetition Maximum (1RM)

Maximal strength will be determined through assessment of the one-repetition maximum (1RM) using the bench press (upper-body) and leg press (lower-body) exercises on four separate occasions (visits 2, 3, 4,and 5). For each assessment, a standardized warm-up consisting of simple stretches and whole-body movements will be completed before performing one set of ten repetitions at 50% of their perceived 1RM. After a rest period of two minutes, participants will perform one set of six repetitions with 70% of their perceived 1RM and then an additional set of four repetitions with 85% of perceived 1RM. From there, one-repetition sets will be completed with progressively increasing loads with two minutes of rest between sets until a 1RM is determined. The 1RM for each exercise will be determined within three to five 1RM attempts to prevent accumulative fatigue from confounding subsequent attempts. Trained investigators will be present during all testing to ensure proper technique is followed for all attempts. Participants will rest for three minutes between determination of their 1RM and completion of the next test.

Muscular Endurance - Repetitions to Failure (RTF)

Muscular endurance will be assessed using the bench press (upper-body) and leg press (lower-body) exercises on four separate occasions (visits 2, 3, 4, and 5). To complete this assessment, study participants will use a load that corresponds to 80% of their Visit 2 1RM for both the bench press and leg press exercises and will be instructed to complete as many repetitions as they can until failure for one set of each exercise. Failure will be defined as resting for more than two seconds throughout any point on the exercise set or technique failure. Total training volume (set x repetitions x load) will be computed and recorded. All subsequent repetitions to failure tests (weeks 2 and 10) will use a load that corresponds to 80% of their visit 2 1RM value.

Wingate Anaerobic Capacity Test (WanT)

Participants will complete a Wingate anaerobic capacity test on four separate occasions (visits 2, 3, 4, and 5) on a Lode Excalibur Sport computerized ergometer (Lode, Netherlands). The testing protocol will begin with five-minute warm-up consisting of light pedaling against zero resistance. The resistance for Wingate testing will be set at 7.5% of body weight (kg) for each participant. Participants will be able to see how much time is left in their warmup and instructed to increase their pedaling speed so they reach their maximal cadence when approximately five seconds remain in the warm-up. Participants will be verbally encouraged to continue pedaling as fast as they can throughout the entire 30-second protocol. Peak power, average power, and time to fatigue will be computed and used as indicators of anaerobic power and fatigue resistance.

Resistance Training Program

Layout of Resistance Training Program

A template of the resistance training program is outlined in Table 2. Participants will be required to complete a daily online checklist of their workout progress and schedule the completion of at least one workout per week under the direct supervision of a research team member inside the Fieldhouse. Intensity will increase throughout the study to ensure a progressive overload to facilitate increases in strength and muscle mass. For the first six weeks (weeks 1-6), each workout will consist of 3 sets of 10 repetitions to momentary muscular failure. The final four weeks (weeks 7-10), 4 sets of 6 repetitions will be completed. One minute of rest in between sets will be allotted for weeks 1-6 while two minutes of rest between each set will be followed for weeks 7-10.

Table 2: Overview of resistance training program

Weeks	Monday, Thursday	Tuesday, Friday
1-6*	Bench press, 3x10<br>Chest flies, 3x10<br>Lat pull, 3x10<br>Seated row, 3x10<br>Shoulder press, 3x10<br>Shoulder shrugs, 3x10<br>Bicep curls, 3x10<br>Triceps extensions, 3x10	Back squat or leg press, 3x10<br>Leg extensions, 3x10<br>Deadlift, 3x10<br>Lunges, 3x10<br>Lying leg curls, 3x10<br>Calve raises, 3x10<br>Ab crunches, 3x25
	Bench press, 4x6	Back squat or leg press, 4x6

7-10‡	Chest flies, 4x6<br>Lat pull, 4x6<br>Seated row, 4x6<br>Shoulder press, 4x6<br>Shoulder shrugs, 4x6<br>Bicep curls, 4x6<br>Triceps extensions, 4x6	Leg extensions, 4x6<br>Deadlift, 4x6<br>Lunges, 4x6<br>Lying leg curls, 4x6<br>Calve raises, 4x6<br>Ab crunches, 3x25

*One minute rest between sets

‡Two minutes rest between sets

Load Progression

In terms of load progression, participants will be asked to perform as many repetitions as they are able on their final set of each exercise. During weeks 1 – 6, if participants are able to complete 12 or more repetitions on their final set, participants will be required to increase the loads they are using to the next level. The final four weeks (weeks 7-10) of the training program, participants will be asked to complete as many repetitions as they are able on their final set. When they are able to complete 7 or more repetitions on their final set, they will be assigned to the next highest load on their progression chart for their subsequent workout. To facilitate proper load progression, four approaches will be employed by our research team. First, participants will be given a progression sheet that will guide them on what loads to use based upon their level of strength for any given exercise. During the first two study visits, this sheet will be reviewed to ensure understanding by the participants. Second, participants will be instructed to complete as many repetitions as they are able on their third and final set. This approach will allow for all participants to auto-regulate their load assignments and when they are able to complete one to two repetitions more than they are programmed to complete, they will be instructed to increase their load. Third, all study participants will be required to perform one workout per week under the direct supervision of a research team member. During this session, discussions surrounding exercise selection, load progression, etc. will be made to ensure participants understand and they are appropriately progressing the loads. Lastly, participants will be told to set a daily workout goal where their total volume-load (sets x repetitions completed x loads used) for each exercise should increase each week. Additionally, participants will be asked two compliance questions during each supervised workout: 1) are they following the load progression instructions as outlined, and 2) do they feel their unsupervised and supervised workouts are different in quality or intensity? These questions will allow for research team members to provide feedback and solutions to maximize consistency between workouts.

Supervision & Compliance to Resistance Training Program

Each resistance training session is anticipated to take approximately 60 minutes to complete. All supervised sessions will be performed between the hours of 0530-0800 hours, 1130-1300 hours, or 1600-2000 hours. Participants will be required to have a fitness staff attendant, personal trainer, strength and conditioning coach or workout partner sign off on completion of each workout. Further, study participants will be required to complete one weekly workout under the direct supervision of a research team member. If desired, participants can complete more supervised workouts per week, but a minimum of one weekly supervised workout will be required. For all unsupervised workouts, study participants must take a photograph of them in the gym with several pieces of exercise equipment in the background. No other human can be in the background of the photo. This photo

must be emailed directly [redacted] will be recorded for compliance.

Dietary Protocol

At the beginning of the study protocol, study participants will be provided dietary instruction from research team members on achieving and maintaining a daily diet that delivers appropriate calories and protein intake. A range of daily caloric needs will be estimated for each study participant by first calculating resting energy expenditure (REE) using both the Mifflin-St Joer and Cunningham equations. These values will be averaged and then this value will be multiplied by an activity factor of 1.55 and 1.75. In addition, study participants will be instructed to maintain a daily protein intake of 1.6 to 2.0 grams of protein per kilogram of body mass each day. Additionally, sample breakfast, lunch, dinner, and snack plans will be provided to all participants to assist them in meeting the calorie and protein intake guidelines for the study. Participants will be required to log their dietary intake over a 3-day period after 0, 2, 6, 8, and 10 weeks of following the supplementation program. For each dietary recall, participants will be asked to enter all of the food and fluid they consume onto a hand-written 3-day food log. Research team members will monitor and analyze all the energy and macronutrient intakes throughout the study and provide recommendations on any required changes if need be. Participants will be provided with instructions on how to properly estimate sample size and be given visual comparators to assist with accuracy of their food entries. If participants fail to meet the dietary recommendations for two consecutive weeks, they will be removed from the study.

Supplementation Protocol

After following the dietary protocol and resistance training program all individuals determined to be weight stable will be randomly assigned to ingest one daily dose in a double-blind fashion of 2 grams of dileucine (Ingenious Ingredients, Lewisville, TX), 2 grams of leucine (NNB Nutrition), or 2 grams of rice flour (placebo). Each dose will be mixed with approximately eight ounces of cold water. On workout days, participants will ingest their assigned dose within 60 minutes of completing their workout. On non-workout days, one dose will be ingested with their morning meal. Participants will be randomly matched according to DEXA fat-free mass for supplementation assignment.

Adverse Event Reporting

The occurrence of adverse events will be recorded throughout the entire duration of the study. When participants visit the laboratory during their weekly supervised workout, participants will report the frequency as well as the severity (‘mild, ‘moderate’, ‘severe’) of any adverse event they report. In addition, adverse events will be collected through spontaneous reporting by the study participants, interaction of a research team member with a study participant, or through review of a study participant’s compliance paperwork.

What forms or instruments will participants complete?

Attach all surveys, questionnaires, screening forms, or data instruments completed by participants (e.g. food diaries, reflection journals, follow-up surveys, etc...). If you are conducting interviews or focus groups, attach a list of applicable questions or prompts.

Note:

- ***Do not attach Recruitment and Consent Materials, will be attached later in the application.***
- ***Qualtrics must be used for survey data collection, unless a prior exception has been granted by the IRB Office. (****Register for Qualtrics here.)*

19.

Diet Log-3-Day-Format.doc

DTS RESISTANCE TRAINING HISTORY.docx

Health Fitness PreParticipation Screening Document.docx

Data Collection - Primary.docx

DTS Data Collection - Training Visit Data Log.doc

DTS Adverse Event Log.docx

EPNL Training Card.xlsx

Exercise Progression.xlsx

DTS Waiver of Documentation of Consent.docx

Participant Screening and Waiver of Documentation of Consent

ASA24

*required

20. **Will you provide compensation to participants?**

No

✓ Yes

*required

Describe your compensation plan:

Note:

- ***Include a description of the amount, timing, and type of compensation.***
- ***Describe who participants who withdraw will be treated. Will they receive full, partial, or no compensation?***

20a. Participants will receive monetary compensation ($300) upon completion of all aspects of the study protocol. To process compensation requests, each participant will need to complete an internal document required for financial processing and a W9. To complete these forms, a participant will need to provide their name, social security number, employment status with Lindenwood University and mailing address. For non-employees of Lindenwood University, compensation will be made in the form of a check that will be made to an address specified by the participant. For Lindenwood University employees, the compensation will be added to their payroll and issued in the same form as how they are paid from Lindenwood University. All payments will be issued upon completion of participation and participants who do not complete the testing protocol may be eligible for a prorated amount of compensation commensurate with the proportion of the study protocol they complete, not to exceed 50% of the available compensation.

*required

Provide a rationale for how the compensation does not pose an undue influence to participants:

An undue influence is an excessive or inappropriate reward, which may affect the voluntariness of a participant's consent and compliance.

20b. Participants in this study will complete five separate visits. Visit 1-5 will only take approximately 1.5 hours. It is estimated that each participant will spend approximately 8 hours of actions directly related to the research study (talking on phone, getting ready for lab visits, driving to lab, completing lab visit) during their supervised training sessions. Participants are being compensated for time spent during the study, but the total compensation will not be enough to unduly influence a participant to complete the study against their personal judgment.

*required

21. **Will your research involve any of the following?**

Check all that apply.

Deception

✓ Investigational Drugs or Dietary Supplements

Investigational Devices

Retaining Data or Biological Samples for Future Research

None of the above

*required

Investigational Drugs or Dietary Supplements

21c.

What drug, substance, or supplement are you investigating? Include:

- *Information about the manufacturer*
- *The ingredients and formulation of the drug, substance, or supplement*
- *A description of any placebo and the constitution of placebo elements*

Leucine (2 grams), Dileucine (2 grams), Rice flower as placebo (2 grams)

*required

Investigational Drugs or Dietary Supplements

21d.

Is this substance considered a "drug" by FDA? The [FDA definition of the term drug] includes, among other things, articles intended for use in the diagnosis, cure, mitigation, treatment, or prevention of disease, and articles (other than food) intended to affect the structure or any function of the body of man or other animals.

✓ No

Yes

*required

Investigational Drugs or Dietary Supplements

21e. *How will you procure, label, handle, and store the drug or supplement?*

All dietary supplements will be procured according to the terms of the research agreement. Alll product will be provided in blinded, labeled containers prior to them arriving in the EPNL. Each beverage will be of a similar volume, texture, and flavor and labeled in indistinguishable bottles to ensure blinding. Upon handling all supplements, research team members will use personal protective equipment (gloves, jackets, eyewear, face mask) when handling all product. Prior to dispensing, the work area will be cleaned with a non-residue surface cleaner. Upon leaving the area, the research team member will discard gloves and use a fresh pair when commencing dispensing of the product.

*required

Investigational Drugs or Dietary Supplements

21f. *How will you dispose of any leftover substances?*

All leftover substances will remain in packaged containers and stored in a cool, dry, dark, and locked location until completion of all study activities. Upon completion, all leftover substances will be discarded with other dry chemicals.

*required

How many participants will you enroll?

---

22. *In most cases, provide a specific minimum number (e.g. 50) of participants to be enrolled. This can at times be a range with the upper limit identified, when it is unsure how many subjects may respond to a survey or how many subjects in a data set may be viable for analysis (e.g. 45-50).*

36

*required

What are the characteristics of your research population?

---

23. *These could include gender, age, ethnicity, race, health status, employment status, etc?* ***If you are recruiting multiple populations, describe by population with anticipated enrollment by population.***

Approximately 36 healthy, resistance trained males between the ages of 18-35 are proposed to complete this study.

*required

What qualifies a participant for your research?

---

24. *Please include all inclusion criteria.*

- Male
- Between the ages of 18-35 years
- Currently participating in resistance training exercise

*required

25. **What disqualifies a participant for your research?**

Please include all exclusion criteria.

- Female
- Participants who are determined to not be weight stable defined as week 0 and week 2 body mass levels deviating by 3% or more.
- Any individual who is currently being treated for or diagnosed with a cardiac, respiratory, circulatory, musculoskeletal, metabolic, immune, autoimmune, psychiatric, hematological, neurological or endocrinological disorder or disease.
- Body mass index > 25 kg/m^2. Individuals with a body mass index greater than 25 kg/m^2, but a body fat percentage less than 25% fat will be accepted into the study
- Individuals who present with any previous injury or illness that would prevent them from appropriately completing all exercise lifts.
- Participants who are not able to fit onto the DEXA table will not be able to participate in the research due to size restrictions of the equipment. This typically includes individuals exceeding 400 pounds or those greater than 6’4”.
- Those individuals with less than 12 months of structured resistance training experience will be excluded from the study.
- Individuals whose maximal relative upper body strength is below 1.0 will be excluded.
- Individuals whose maximal relative lower body strength is below 1.5 will be excluded.
- All participants who DO NOT abstain from taking any additional forms of nutritional supplementation deemed to be ergogenic or that will impact resistance training adaptations (pre-workouts, creatine, beta-alanine, etc.) for four weeks prior to beginning this study and for the entire duration of the study.
- Individuals who are currently using or have used within the past 12 months anabolic-androgenic steroids.

*required

26. **Are any of the following an exclusion criterion?**

Check all that apply:

✓ Age

✓ Gender

Race or Ethnicity

Language

Not applicable

*required

27. **Provide a justification for how this exclusion does not adversely affect the rights and welfare of participants:**

Those individuals less than 18 and greater than 35 years of age will be excluded. Participants younger than 18 are excluded due to necessity of parental consent. Participants greater than 35 years old are excluded for two reasons. First, the primary demographic of our society which resistance trains and will consider using various dietary supplements to augment their training adaptations fall within this range. Second, as males reach 35 years of age, circulating levels of androgens begin to decrease which is associated with declines in strength, power, and muscle size. Additionally, known influences of female sex steroid hormones impact how strength and body composition adaptations occur. Due to this study being an initial pilot, females are excluded to avoid the confounding influence of changes in female sex steroid hormones across the menstraul cycle.

*required

28. **Will you be enrolling any of the following populations?**

Check all that apply:

Minors

Pregnant Women or Fetuses

Prisoners

✓ Not applicable

*required

29. **Are you enrolling any vulnerable populations?**

Check all that apply:

Individuals with Impaired Decision-Making

Economically Disadvantaged

Educationally Disadvantaged

✓ Not applicable

*required

31. **Are you enrolling participants with special cultural considerations or who reside in a different country?**

✓ No

Yes

*required

32. **Is anyone on the research team in a position of authority over participants?**

This type of relationship could include, but is not limited to, the following: supervisor/staff, teacher/student, coach/team members, counselor/client.

No

✓ Yes

*required

32a. **How will the recruitment process ensure participants do not feel coerced into participation?**

Coercion occurs when someone feels overtly or implicitly obligated to participate based on some aspect of their relationship with the researcher.

It is anticipated that study participants will be recruited from within and outside the Lindenwood University community. However, faculty membersat Lindenwood

Universitymay have students in his/herclasses that are participants in this studyin which a research requirementwill be required as part of therequirements for theirclass.In these situations, all students have four options to receive credit for this assignment: a) observe data collection, b) participate in a study, c) attend a scientific conference, or d) attend a thesis proposal or defense. Thus, any study participant who is also a student in a faculty member'sclass will have the same opportunities to complete this requirement as all other students. If the study participant elects to not participate in this study, they have other means to earn credit for this class requirement. This requirement is clearly outlined in each of the relevant course syllabi.

*required

33. **How are you recruiting participants?**

Check all that apply:

- Lindenwood Participant Pool
- Participant Registry or Database
- ✓ Flyer
- Telephone Script
- ✓ Email Script
- ✓ Social Media Script
- Website
- ✓ Classroom Presentation
- ✓ Other Recruitment Method(s)

33b. **Flyer Recruitment**

*required

Where will you post flyers?

33b-1. *If posting flyers at Lindenwood University, please indicate the specific buildings in which flyers will be posted.*

Lindenwood University and public places within the St. Charles area (such as grocery stores and gymnasiums). Lindenwood buildings may include the Field House, Spellman Center, Evans Commons, and the Library.

*required

Does posting flyers require approval?

33b-2. *Other sites may or may not require permission. You are required to ensure compliance with the requirements at each site.*

Note: All posting of flyers on Lindenwood University sites requires prior permission. Approval from the Dean or building coordinator for each area must provide approval for the posting of research recruitment materials in their area.

✓ No

Yes

*required

33b-4. Attach all flyers you will use for recruitment.

[Dileucine Training Study Flyer.pdf]

*required

33d. **Email Script**

---

*required

33d-1. How will you obtain email addresses?

Through initial contact initiated by study participant. Participants may call or email the lab and express interest.

*required

Does your email recruitment process require prior authorization or approval?

33d-2. Note: Lindenwood University will not release student email addresses for research purposes. If you plan to recruit subjects on the LU campus via email, please contact the Dean or supervisor most closely related to those subjects for permission for them to distribute your recruitment email at their discretion.

✓ No

Yes

33d-3. Attach a record of authorization for use of emails for research recruitment purposes (such as an email or formal approval).

33d-4. Attach the script you will use to recruit participants through an email.

DTS EMAIL SCRIPT.docx

*required

33e. **Social Media Script**

*required

Where will you post information, and how do Terms of Service apply to this posting?

33e-1. *Researchers are required to be aware of the Terms of Service and expectations for posting in social media or online spaces. As these Terms of Service may change over time, researchers may need to modify this section of the application as these terms or expectations change.*

Information about the study will be posted on the EPNL's Instagram, Twitter, and Facebook page.

33e-2. Attach the script you will use to recruit participants over social media.

SOCIAL MEDIA SCRIPT-2021.01.22.docx

*required

32g. **Classroom Presentation**

*required

33g-1. Where and when will this take place?

This will take place at Lindenwood University. It will take place during classes when permission is given by the professor.

*required

33g-2. Does this presentation require approval?

✓ No

Yes

*required

33h. Other Recruitment Method(s)

*required

33h-1. Describe your recruitment method:

Through Lindenwood's online platform InvolveU

Attach any relevant script or approval for this recruitment method.

33h-2. SOCIAL MEDIA SCRIPT.docx

Dileucine Training Study Flyer.pdf

*required

34. **Does your research involve any of the following?**

Please contact the IRB Office if you have questions about making this decision. We are happy to assist in helping you select your review pathway.

Sensitive and Identifiable Information

✓ • Your research is collecting data which fall under the LU IRB definition of Sensitive Data. You will also be collecting and retaining Identifiable Data.

Physical, Pharmacological, or Psychological Intervention

✓
- You will be performing physical procedures such as, but not limited to, drawing blood or collecting tissue, having participants ingest nutritional supplements or performing an exercise bout or exercise training program.

  OR

- You will be performing behavioral procedures that may affect the behavior, mood, or ability of participants. These procedures are *not* brief in duration, harmless, or painless; they are potentially physically invasive; they may potentially have a significant adverse lasting impact on the subjects; or they may be offensive or embarrassing to participants.

Vulnerable Populations

- Some or all of your participants are likely to be vulnerable to coercion or undue influence, such as children, prisoners, individuals with impaired decision-making capacity, or economically or educationally disadvantaged persons.

None of the above

- If your study involves minors, you may select this option only if your research is conducted in established or commonly accepted educational settings, involving normal education practices.

Consenting Adult Participants

*required

How will you obtain informed consent from adult participants?

35. *If multiple populations are consented, describe the different process and consent form used for each population when applicable. In thinking through your consent process, please describe:*

- *Where you will consent participants, which should be a private area accessible to researchers and participants.*
- *When you will consent participants, which should include time for participants to consider the research, ask questions, and consult with family, friends or physicians. The timing of the consent process should not cause undue inconvenience or hardship to participants.*
- *How the consent process will take place, including any issues you anticipate in terms of the vulnerability or accessibility of potential participants.*

Most commonly, participants will be consented inside the Exercise and Performance Nutrition Laboratory. When possible, interested participants will be provided a consent form via email to give them more time to review and formulate questions. All participants will be given as much time as necessary to formulate questions and consult family, friends, physicians, and whomever else they would like. Prior to any data collection and at a time they schedule, potential study participants will, after having their questions answered, sign and date the consent form.

*required

Are you requesting a Waiver of Documentation of Consent?

36. *A Waiver of Documentation of Consent is used when:*

- *You are providing an Informed Consent Document to participants, but will not be obtaining their written signatures.*

- *Reading an Informed Consent script verbally, or over the phone, to participants when conducting a screening conversation and sharing study requirements.*

No

✓ Yes

*required

36a. *Select one of the following and then provide a rationale explaining why this Waiver of Documentation of Consent will not adversely affect the rights and welfare of participants:*

✓ The only record linking the subject and the research would be the consent document, and the principal risk would be potential harm resulting from a breach of confidentiality. Each subject will be asked whether the subject wants documentation linking the subject with the research, and the subject's wishes will govern.

*required

36a-1. How will the rights and welfare of participants be protected, even though you will not be obtaining documentation of informed consent from participants?

After the phone or email screen, any and all gathered information will be kept in a secure and locked filing cabinet behind two locked doors. Once participants arrived to the EPNL, any further questions the participant may have will be answered and full consent will take place at that time. No harm should come from asking participants to complete an overnight (8 - 10 hour) fast. Most people from the time of their last meal (dinner) until the time of their first meal (breakfast) the next day will have gone through the same overnight fast, if not longer. Furthermore, several studies have shown that ~20 - 30% of adults will all-together skip breakfast (St. Onge et al. 2017; Greenwood 2007; Sungsoo et al. 2003; Ruxton 1997; Haines 1996) [References attached]

The research presents no more than minimal risk of harm to subjects, and involves no procedures, for which written consent is normally required outside of the research context.

*required

Are you requesting a Waiver of Consent or will you be altering or removing any of the required Elements of Consent?

37.

A Waiver of Consent is used when you will not be obtaining any informed consent from participants. This is rare occasion, typically used when a signature would normally be required, but contacting each potential participant would be impracticable.

✓ No

Yes

Attach all of your Adult Informed Consent Documents:

38.

All current Lindenwood University Research Informed Consent Documents and Information Sheets are available here. These templates include instructions. Sections indicated in these templates may not be revised or altered by researchers.

DTS - Consent Form.12.3.20 (2).docx

DTS - Consent Form.01.20.21.docx

Consenting Minor Participants

*required

Are you enrolling minor participants?

39.

A minor is defined as a participant under the age of 18 at the time of enrollment. If research is being conducted in an educational environment, please visit our guidance for K-12 research.

✓ No

Yes

Other Participant Considerations

*required

40. **Are you enrolling Prisoners?**

✓ No

Yes

*required

41. **Are you enrolling Pregnant Women, Neonates, or Fetuses?**

✓ No

Yes

*required

42. **Are you enrolling any participants who by virtue of a cognitive, health, social or other reason may not be able to provide informed consent?**

✓ No

Yes

*required

43. **Are you enrolling participants who do not speak English, or who may not have English proficiency?**

✓ No

Yes

*required

Are you collecting Personally Identifiable Information?

According to [Lindenwood University IRB policy], this could include:

44.

- *Any data element which singly or in combination could be used to directly identify a participant. The LU IRB uses the list HIPAA identifiers as a helpful guide.*
- *Any combination of variables in a data set that might permit the indirect identification of a participant.*
- *Any element of the research design, such as timing or location, that may permit the incidental identification of participants.*
- *Any code linking a participant and their data.*

No

✓ Yes

*required

44a. *Check all that apply:*

✓ Location information (address, geotagged data, etc...)

✓ Dates related to an individual (birth date, graduation date, dates of service, etc...)

✓ Phone numbers

✓ Email addresses

✓ Social Security Numbers

IP Addresses

Biometric Identifiers (finger or voice print, unique biomarkers, etc...)

Photographs, video, or audio

Other:

*required

45. **How will you maintain participant privacy and confidentiality?**

Describe all protective measures taken while collecting, recording, handling, and storing research data. Provide details on any methods used to deidentify, code, or transfer data.

Only information required to accurately complete all aspects of testing in this study will be collected. Participants will be kept anonymous during data collection, storage, and release by all research team members following standard confidentiality protocols. All data will be de-identified by assigning each participant a unique, random identification number. Hard copy data will be kept in a locked filing cabinet inside of the Exercise and Performance Nutrition Laboratory, which is locked when not occupied by staff/student workers. All electronic data is password protected. All electronic data will be kept on a password protected computer for no longer than 3 years. All data from participants who do not qualify will be destroyed. All biological samples transferred elsewhere for analysis will only be labeled with the participant's unique, random identification number.

*required

46. **What technological and physical safeguards will you use to protect data from inappropriate use or disclosure?**

Check all that apply:

Anonymizing data at point of collection (e.g. using Qualtrics to anonymize data)

✓ Locked room or space

✓ Behind a double lock (e.g. locked cabinet in a locked room)

✓ Restricted access to authorized research team members

✓ Password-protected computer or device

Password-protected folder or storage

Encrypted file transfer

✓ Secure Lindenwood University Research Drive (if collecting PHI or Sensitive Data)

Destruction of source data immediately after processing

Destruction of audio or visual data immediately after transcription

Modification of audio or visual data to eliminate identifiers

Other:

*required

47. **What will you do with data or specimens at the conclusion of the study?**

Check all that apply:

I am not collecting any identifiers. I will retain data for the required retention period (3 years, or longer as required by other agencies) and then destroy it.

I will deidentify data or specimen logs and erase or destroy any related codes. I will retain data for the required retention period (3 years, or longer as required by other agencies) and then destroy it.

✓ I will keep identifiable data for the required retention period (3 years, or longer as required by other agencies) and then destroy it.

I will destroy any leftover specimens.

I will retain data and specimens for future use.

Other:

*required

48. **Will you make the results of the research available to participants or others?**

Please indicate if you will make any form of the research data available, such as through publication, presentation, or thesis/dissertation publication.

No

✓ Yes

*required

48a. *Describe how you will share results, with attention to the potential violation of participant privacy or disclosure of sensitive information about any stakeholders:*

No data will be shared via email or phone. No data will be shared until an individual has finished the study or it has been deemed by researchers that the data request will not compromise the integrity of the study. All requests for data will be granted in person and only given to the individual who completed the study.

Describing Risks

*required

What are the potential risks related to your research?

49. *All research poses potential risk to participants or others. In many cases, these risks are clearly identifiable. But even in relatively simple data collection or survey research, subjects are being exposed to potential informational risks. Visit our [guidance on identifying risks in research] for more guidance as you complete this section.*

✔ Privacy Risks

*required

Describe any and all privacy risks:

49a. *Provide detail regarding the frequency, severity, and duration of each risk.*

Loss of privacy and confidentiality with exchanging contact information and private information

*required

How will you minimize these risks?

49b. Participant data will be de-identified with a study code identifier for all data collection and reports. All data will be stored on in a locked filing cabinet inside of the Exercise and Performance Nutrition Laboratory, which is locked when not occupied by staff/student workers. All electronic data is password protected.

Social or Psychological Risks

✔ Physical Risks

*required

Describe any and all physical risks:

49e. *Provide detail regarding the frequency, severity, and duration of each risk.*

-Exposure to radiation through the completion of a DEXA.-BIA assessment poses a near trivial level of risk for someone with a pacemaker or internal defibrillator.-Risks of upset stomach or GI distress from -Risk of exposure to COVID-19 for participants, research team members, and others on Lindenwood's campus who may come in contact with them.-Risk of privacy during Ultrasound-Risk of injury from performance testing and/or resistance training program

*required

How will you minimize these risks?

49f. Radiation exposure is limited through having trained investigators complete all DEXAs as to minimize or eliminate the need for subsequent scans by the DEXA. All research team members operating the DEXA will have completed an internal training program and be indicated on a delegation of authority log of this ability. Risk of contracting and spreading COVID-19 will be minimized by the development and following of the EPNL's "COVID-19 Exposure Control SOP" as well as following guidelines set forth by Lindenwood University. Our exposure control plan takes into account social distancing, rearranging the lab to allow for more space so team members and participants can spread out more efficiently, COVID-19 training documents, required PPE to be worn, and tracking symptoms for both team members and participants. Supplementation: Ingesting amino acids may cause upset stomach or gastrointestinal distress, although the most common occurrences from this happen with larger doses of leucine and amino acids.Physical Privacy during Ultrasound: The ultrasound technique will be complete on the muscles in the front of your thigh on your dominant leg. To complete this assessment, research team members may need to roll up your shorts on that leg. To minimize this risk, ultrasound tests will be completed in an area of the laboratory where exposure to other people will be limited. While injury may occur during exercise, this is not likely due to all tests and exercises you are asked to perform are of an intensity presumed to be completed by individuals of good health and active fitness levels on a weekly basis.

Risks to third parties (institutions, community, researchers, or non-consented individuals)

Other:

*required

50. **Does your study involve collection of blood, tissue, or biological samples?**

✔ No

Yes

*required

51. **Will your research have a Data and Safety Monitoring Plan?**

A Data and Safety Monitoring plan is typically required for clinical research, or research involving intensive physical or biological interventions.

✓ No

Yes

51c. **Attach any applicable documents:**

General Benefits

*required

Describe the potential for benefits to society and your field of study:

52. This question regarding general benefit is related to your answer in Question 15, where you discussed the importance of this research for your field of study.

No direct benefits will result from this study protocol. Our research findings will contribute to a greater understanding between the training adaptations of leucine and dileucine after a 10 week supplementation regimen. This information will allow researchers, food/supplement companies, coaches, and trainers to improve the quality and efficacy of leucine-containing products given during a training session. Notably, development of such products may help improve the dosing and delivery of key amino acids for all populations, particularly the aged and elderly.

Direct Benefits to Participants

*required

53. *Describe any potential for direct benefits to participants (A direct benefit accrues to the participant from a research intervention. Testing, scans, or measurements taken during the research or compensation cannot be considered a direct benefit):*

No direct benefits to participants are anticipated.

Your Attachments

This section provides an overview of documents you have attached when prompted by the application. You may also use this section to add or delete documents prior to submission, or to ensure that you have uploaded all required documents. Please note:

- This section is primarily to review documents you have attached when prompted by the application.
- If you have followed the attachment prompts in the application, you will not need to upload any additional documents in this section.
- You may not need to attach any documents to many of these areas.

Non-LU Research Team Member Training Certificate

Site Permission Attachment

Conflict of Interest Form

Current Scholarship

REFERENCES.docx

Participant Data Collection Instruments

Diet Log-3-Day-Format.doc

DTS RESISTANCE TRAINING HISTORY.docx

Health Fitness PreParticipation Screening Document.docx

Data Collection - Primary.docx

DTS Data Collection - Training Visit Data Log.doc

DTS Adverse Event Log.docx

EPNL Training Card.xlsx

Exercise Progression.xlsx

DTS Waiver of Documentation of Consent.docx

Participant Screening and Waiver of Documentation of Consent

ASA24

Site Flyer Approval

Phone Recruitment Script

Email Recruitment Script

DTS EMAIL SCRIPT.docx

Email Recruitment Authorization

Social Media Recruitment Script

SOCIAL MEDIA SCRIPT-2021.01.22.docx

Website Recruitment Script

Classroom Presentation Recruitment Approval

Other Recruitment Script

SOCIAL MEDIA SCRIPT.docx

Dileucine Training Study Flyer.pdf

Flyer Recruitment Document

Dileucine Training Study Flyer.pdf

Informed Consent Documents

DTS - Consent Form.12.3.20 (2).docx

DTS - Consent Form.01.20.21.docx

Exempt Information Sheets

Assent Form Documents

Parental Consent on Behalf of Minor Documents

Data and Safety Monitoring Plan

PPSRC

Additional Documents

Only attach documents in this area if required by the IRB.

EPNL Hydration Protocol.docx
