# Supplementary material for: Dileucine ingestion, but not leucine, increases lower body strength and performance following resistance training: A double-blind, randomized, placebo-controlled trial
Source: PLoS One. 2024 Dec 31;19(12):e0312997. doi: 10.1371/journal.pone.0312997 (PMC11687731; doi:10.1371/journal.pone.0312997)
Supplement: Attachment — Submitted filename: Hagele-PONE-D-24-07083R1-Response to Reviewers-10.04.24.docx [file pone.0312997.s004.docx]

COMMENTS:

1. In the work, all unit entries (text, tables) have still not been corrected in accordance with the reviewer's comments - they should be unified everywhere, e.g. "kcal∙kg^-1^" instead of "kcal/kg"; "W∙kg^-1^" instead of "W/kg”, „g∙kg^-1^∙day^-1^” instead of „g/kg/day”, „kg∙m^-2^” „kg/m^2^” etc.

**Author Response: Thank you for your feedback. We have now carefully revised and unified all unit entries throughout the manuscript, ensuring consistency across both the text and tables. All units have been updated to the correct scientific notation per your suggestion.**

2. Line 60 - insert a space between (6-8%) and [14,15].

**Author Response: Thank you for pointing this out. We have inserted the space between "(6-8%)" and the citation "[14,15]" as requested.**

3. line 70: first use - "DiLEU (DILEU)", - use the full name and abbreviations can be used in the rest of the text.

**Author Response: Thank you for your suggestion. We have now provided the full name at its first mention followed by the abbreviation in parentheses, and used the abbreviation throughout the remainder of the text.**

4. Line 258 – change „caloric” to „energy”.

**Author Response: Thank you for your suggestion. We have replaced “caloric” with “energy” throughout the manuscript as requested.**

5. Table 2 - The lack of statistical values makes it impossible to assess whether the participans did not differ "at the entrance" to the individual groups (LEU vs. DILEU vs. PLA). This point should be addedd so as not to leave readers with potential doubts.

**Author Response: Thank you for your observation. We have now added the relevant statistical values to Table 2 to clarify that there were no significant differences between the groups at baseline.**

6. Table 2 - In the unit description for Relative Leg/Bench Press, put "Body Mass" in the subscript.

**Author Response: Thank you for the comment. We have revised the unit description in Table 2 to include “Body Mass” in the subscript as requested.**

7. Table 3 – insert „intake” after „Relative Energy”.

**Author Response: We have inserted “intake” after “Relative Energy” in Table 3**

8. Table 4 – insert „mass” after "Dry lean".

**Author Response: We have inserted “mass” after “Dry lean” in Table 4**

9. In the revised description of the results, there are probably mistakes in some points - these descriptions do not match the data from the tables.

- In the line 359 - the authors write "a significant increase in bench press 1RM for DILEU compared to PLA (p = 0.02; 95% CI: 6.8, 75.9 kg)" - here it should rather be "total strength" (see table 5).

- in the 374 - similarly as above. Authors write „…increase in leg press RFT” - and it should be " …increase in total reps..".

- The whole manuscript should be checked in this respect to make sure all descriptions are correct.

**Author Response: Thank you for identifying these discrepancies. We have corrected the descriptions in the highlighted lines and thoroughly reviewed the entire manuscript to ensure that all results descriptions are accurate and match the data in the tables.**

10. Lines 383 and 384 - p values are incorrect and there is an error in the descriptions („p = 0.0.54”;  „p = 0.0.65” and „p = 0.0.92”). This should be corrected and the entire text/tables/figures should be checked again.

**Author Response: Thank you for pointing this out. We have corrected the p-values in lines 383 and 383 and reviewed the entire manuscript, including text, tables, and figures, to ensure all p-values are accurate and correctly reported.**

11. Line – „calories” are not consumed - it is de facto a unit. Please correct the sentence and use "energy intake".

**Author Response: Thank you for the suggestion. We have corrected the sentence to use “energy intake” instead of calories to accurately reflect the concept.**
